# Case Report: A novel homozygous splice-site variant in the *C3* gene causing complete complement C3 deficiency in two unrelated Moroccan patients

**DOI:** 10.3389/fimmu.2026.1860941

**Published:** 2026-06-29

**Authors:** Chaymae Oujane, Mohamed Hbibi, Ibtihal Benhsaien, Aicha Naitobrah, Nassima Akhrichi, Zahra Aadam, Hajar Kalil, Jalila El Bakkouri, Ahmed Aziz Bousfiha, Fatima Ailal

**Affiliations:** 1 Laboratory of Clinical Immunology, Infection And Autoimmunity (LICIA), Faculty of Medicine and Pharmacy, Hassan II University of Casablanca, Casablanca, Morocco; 2 Departement of Pediatric Hematology-Oncology, SHOP, CHU Hassan II, Fez, Morocco; 3 Department of Clinical Immunology and Pediatrics Infectious diseases, Abderrahim El Harouchi Mother Child Hospital, Ibn Rochd University Hospital Center, Casablanca, Morocco; 4 Immuno-Serology Hospital Laboratory, Ibn Rochd University Hospital, Casablanca, Morocco

**Keywords:** C3 deficiency, complement deficiency, founder effect, inborn errors of immunity, Morocco, primary immunodeficiency, recurrent infections

## Abstract

Complement component 3 (C3) plays a central role in innate immunity as a convergence point of the classical, alternative, and lectin pathways. Complete C3 deficiency is an extremely rare inborn error of immunity, typically associated with recurrent severe infections and, in some cases, immune complex–mediated or autoimmune manifestations. Despite its clinical significance, fewer than 50 cases have been reported to date, and the genetic and phenotypic spectrum remains incompletely defined. We report two unrelated pediatric patients originating from the same geographic region in Morocco, both presenting with recurrent infections. Comprehensive clinical evaluation, immunological workup including complement assays, and genetic analysis using next-generation sequencing (NGS) were performed. In silico tools were used to predict the functional impact of the identified variant, and familial segregation analysis was conducted. Both patients presented with chronic and recurrent infections, predominantly affecting the respiratory and otorhinolaryngological systems, without identification of consistent pathogens. Immunological investigations revealed normal lymphocyte subsets, immunoglobulin levels, and neutrophil oxidative burst. In contrast, complement analysis showed undetectable C3 levels and markedly reduced CH50, with normal C4, consistent with isolated complete C3 deficiency. NGS identified a novel homozygous splice-site variant in the C3 gene (NM_000064.3:c.4030-1_4030delinsCT) in both patients. The variant is absent from population databases and predicted to severely disrupt normal splicing, likely leading to loss of function. Segregation analysis confirmed heterozygous carrier status in the parents, supporting autosomal recessive inheritance. Notably, both patients originate from the same geographically confined and consanguineous region, suggesting a possible founder effect.We report two cases of complete C3 deficiency associated with a novel homozygous splice-site variant, expanding both the clinical and genetic spectrum of this rare condition. Our findings highlight that C3 deficiency may present with non-severe but chronic infections and emphasize the importance of complement evaluation in unexplained infectious phenotypes. The geographic clustering of cases raises the possibility of a founder mutation, warranting further population-based genetic studies.

## Introduction

Inborn errors of immunity (IEIs) constitute a clinically and genetically heterogeneous group of disorders affecting the development, function, or regulation of the immune system (1). Among these, complement deficiencies represent a distinct category that accounts for approximately 5% of all IEIs worldwide (2, 3). The complement system, comprising more than 50 soluble and membrane-bound proteins, plays a pivotal role in innate immunity through opsonization, pathogen lysis, clearance of immune complexes and apoptotic debris, and modulation of adaptive immune responses (3–5). Complement component 3 (C3) occupies a central position within the complement cascade, acting as a convergence point for the classical, alternative, and lectin pathways. Its activation leads to the generation of key effector molecules, including C3b, which serves as the principal opsonin, and C3a, an anaphylatoxin with pro-inflammatory activity (6). Consequently, complete C3 deficiency (OMIM #613779), caused by biallelic loss-of-function variants in the *C3* gene, results in a profound and global impairment of complement activation, abrogating all downstream effector functions simultaneously.

Since its first description in 1972 in a South African patient with repeated pyogenic infections and serum C3 levels of less than 1% of normal (7), fewer than 50 cases have been reported worldwide (8). This extreme rarity, combined with considerable phenotypic heterogeneity ranging from severe early-onset invasive infections to milder chronic infectious patterns and variable autoimmune complications, frequently contributes to delayed or missed diagnoses, particularly in settings with limited access to specialized immunological investigations (8, 9). The clinical phenotype of complete C3 deficiency classically manifests as markedly increased susceptibility to recurrent and severe infections, particularly those caused by encapsulated pyogenic bacteria such as *Streptococcus pneumoniae*, *Haemophilus influenzae* type b, and *Neisseria meningitidis* (10, 11). Additionally, approximately 26% of patients develop immune complex-mediated diseases and autoimmune manifestations, most commonly membranoproliferative glomerulonephritis and systemic lupus erythematosus (SLE)-like syndromes, reflecting the critical role of C3 in immune complex clearance and maintenance of self-tolerance (10, 11).

From a genetic standpoint, C3 deficiency is inherited in an autosomal recessive manner and is caused by a diverse array of pathogenic variants, including missense (12), nonsense, splice-site mutations (13), compound heterozygous combinations (14, 15), large deletions (16, 17), and homozygous splice-site variants disrupting mRNA processing (18). Despite the expanding catalog of disease-causing variants, genotype-phenotype correlations remain poorly defined, partially because of the extreme rarity of the condition. Moreover, clinical heterogeneity within families carrying identical variants underscores the contribution of additional genetic, environmental, and stochastic factors to disease expression (8, 19). In populations with high rates of consanguinity, as found in North Africa and the Middle East, rare autosomal recessive disorders are more prevalent, and the occurrence of geographically clustered cases may indicate the presence of founder mutations (20, 21). In this context, the identification and characterization of novel variants are essential not only for accurate molecular diagnosis but also for improving genetic counseling, enabling targeted carrier screening, and elucidating the epidemiology and pathophysiology of rare genetic disorders.

In this report, we present the detailed clinical, immunological, and molecular characterization of two unrelated pediatric patients originating from the same consanguineous region in northern Morocco, both found to have complete C3 deficiency attributable to a novel homozygous splice-site variant in the *C3* gene. These cases expand the molecular and phenotypic spectrum of C3 deficiency and provide compelling evidence for a founder mutation in the Taounat region of Morocco.

## Methods

### Patients and clinical assessment

Both patients were evaluated and followed at the Department of Pediatric Infectious Diseases and Clinical Immunology, Mother-Child Hospital Abderrahim El Harouchi, Ibn Rochd University Hospital, Casablanca, Morocco. Detailed clinical evaluations were performed, including comprehensive medical history, physical examination, and relevant laboratory investigations. Family pedigrees were obtained through direct interviews with the parents. Written informed consent was obtained from the patients’ legal guardians in accordance with the Declaration of Helsinki.

### Immunological testing

Serum complement activity was assessed by CH50 using an ELISA-based technique, and alternative pathway activity was measured by AP50. Serum C3 and C4 levels were quantified by turbidimetry. Lymphocyte subset analysis was performed by flow cytometry using monoclonal antibody panels targeting CD3, CD4, CD8, CD19, and CD16/CD56. Serum immunoglobulin levels (IgG, IgA, IgM) were measured by nephelometry. The results interpreted using age-matched reference ranges.

### Genetic analysis

Genomic DNA was extracted from peripheral blood leukocytes using the Invitrogen™ PureLink™ Genomic DNA Mini Kit (Ref: K182001). Targeted next-generation sequencing (NGS) was performed using a custom gene panel covering genes associated with inborn errors of immunity, including complement pathway genes. Sequencing libraries were prepared using a hybridization capture-based approach and sequenced on an Illumina platform. Sequence reads were aligned to the human reference genome (GRCh37), and variants were called and annotated according to the standard guidelines of the Human Genome Variation Society (HGVS). Variants were further filtered based on allele frequency in population databases (gnomAD, 1000 Genomes) and the population frequency of variant was verified using the GeneBe platform (https://genebe.net/). Splicing impact was assessed using Alamut Visual Plus v.2.0, which integrates multiple splice prediction algorithms, complemented by SpliceAI for deep learning-based splice disruption scoring. Transcript annotation and exon boundary verification were performed using Ensembl (https://www.ensembl.org/). Variant pathogenicity was classified according to ACMG/AMP standards (22, 23).

## Results

### Case description

#### Patient 1

**Figure 1:**
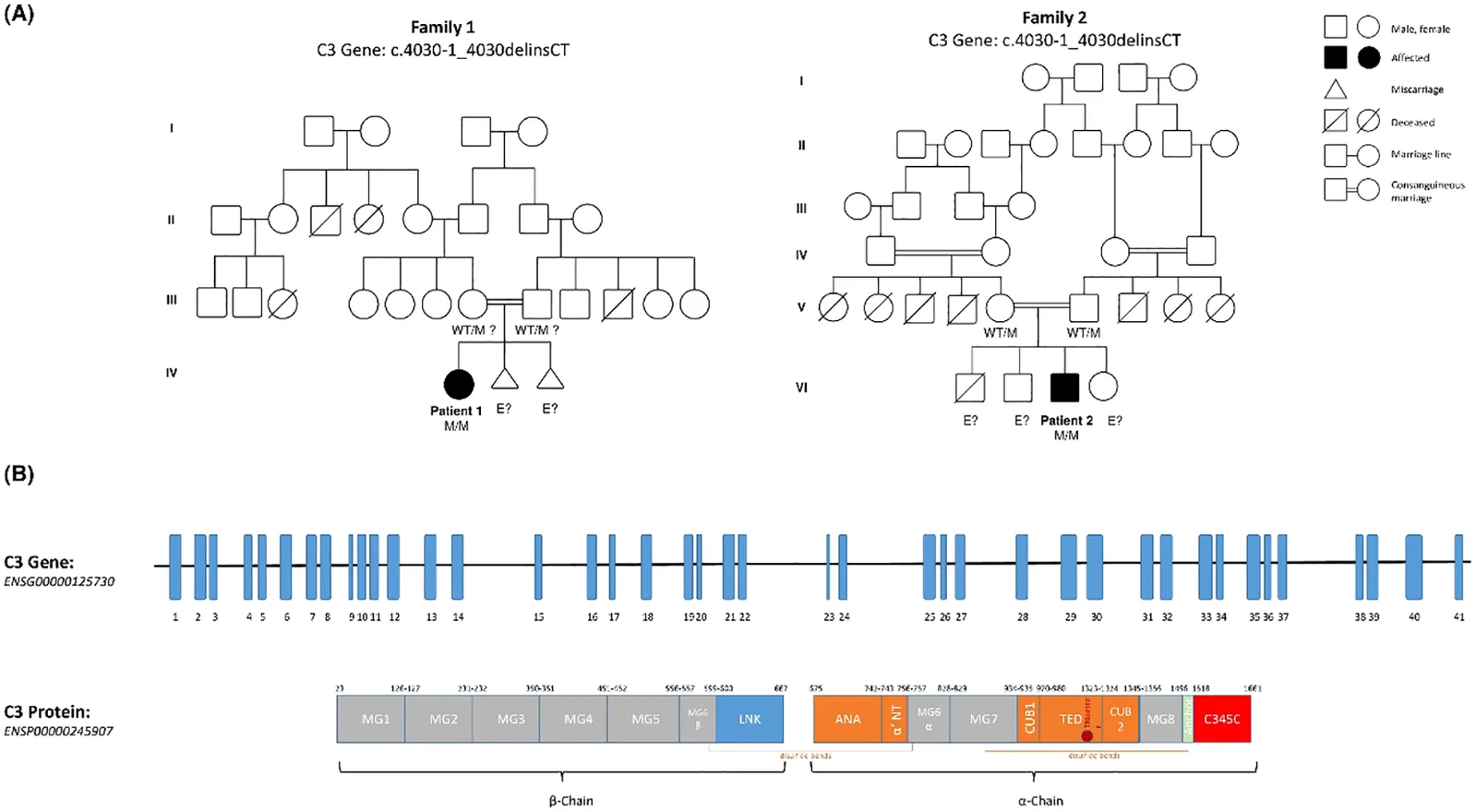
Pedigrees of the two families and schematic representation of the C3 gene variant location. **(A)** Family pedigrees of Patient 1 (Family 1) and Patient 2 (Family 2) are presented. Filled symbols indicate affected individuals (M/M: homozygous mutant). Symbols with a diagonal slash represent deceased individuals. Half-filled or carrier-designated symbols (WT/M) indicate confirmed or suspected heterozygous carriers. Triangles represent miscarriage. E?: genotype not determined. Double horizontal lines between couples denote consanguineous unions. **(B)** Schematic representation of the C3 gene exon structure (top) with the variant position indicated. The lower diagram illustrates the domain architecture of the mature C3 protein, comprising the β-chain (MG1–MG6β, LNK) and the α-chain (ANA, α’NT, MG6α, MG7, CUB1, TED, CUB2, MG8, C345C). The red dot marks the location of the splice-site variant c.4030-1_4030delinsCT at the acceptor site of exon 32, within the region encoding the C-terminal C345C domain.

We report a 4-year-old female patient who was referred for evaluation of recurrent infections. The patient was born to first-degree consanguineous parents originating from the Taounat province in northern Morocco. She was the product of the mother’s second pregnancy, which was complicated by preeclampsia, and was delivered via cesarean section at term. The postnatal period was unremarkable, with normal adaptation and age-appropriate psychomotor development. The patient received complete vaccination according to the Moroccan national immunization schedule. Family history was notable for two maternal miscarriages occurring at approximately three months of gestation and the death of a paternal uncle at 13 years of age from an undetermined cause (**Figure 1A**).

The clinical presentation began at 10 months of age with recurrent febrile episodes accompanied by chronic wheezing. From the age of 2 years, she was empirically managed as having bronchial asthma with bronchodilator therapy. During the same period, the patient developed recurrent purulent otorrhea requiring multiple courses of antibiotic therapy and several hospitalizations. Her infectious course was further complicated by an episode of facial cellulitis secondary to complicated acute otitis media, which was successfully treated with amoxicillin-clavulanic acid. No episode of bacteremia, meningitis, or deep tissue infection was documented.

At the time of referral, the patient was alert, conscious, afebrile, and hemodynamically stable, with a weight of 17 kg (−1 SD for age) and a height of 106 cm (appropriate for age). Otorhinolaryngological examination demonstrated active right-sided purulent otorrhea, while the left ear appeared normal. The oropharynx, lymph nodes, lungs, cardiovascular system, and abdomen were unremarkable (**Table 1**).

**Table 1:** Clinical characteristics of the two patients.

Parameter	Patient 1	Patient 2
Sex/Age	Female/4 years	Male/8 years
Geographic origin	Taounat region	Taounat region
Consanguinity	First-degree	Third-degree
Pregnancy/Birth	Preeclampsia, cesarean section	Uneventful
Growth at evaluation	Weight (−1 SD); height normal	Normal
Vaccination status	Complete (national schedule)	Complete (national schedule)
Family history	Miscarriages, uncle deceased	Multiple early deaths
Age at onset	10 months	1 month
Respiratory symptoms	Chronic wheezing	Respiratory distress
ENT infections	Recurrent purulent otorrhea; facial cellulitis (complication of acute otitis media)	Recurrent purulent otorrhea; recurrent tonsillitis; pharyngeal abscess
Other infections	–	Urinary infection
Autoimmune features	IgA vasculitis	IgA vasculitis
Other symptoms	–	Headache, edema, purpura

ENT, ear, nose, and throat; SD, standard deviation.

Laboratory investigations showed microcytic hypochromic anemia (hemoglobin 10.8 g/dL, MCV 77 fL, MCH 26 pg) and negative HIV serology. Lymphocyte immunophenotyping was within normal ranges: CD3 2,922/mm3, CD4 1,395/mm3, CD8 1,217/mm3, CD19 955/mm3, and NK cells (CD16/CD56) 164/mm3. Serum immunoglobulin levels were normal (IgA 1.09 g/L, IgG 9.19 g/L, IgM 1.91 g/L, IgE 94.52 IU/mL). The dihydrorhodamine (DHR) test was 94.23%, within the normal range, excluding a major defect in neutrophil oxidative burst. In contrast, complement evaluation revealed completely undetectable quantitative C3 (<0.01 g/L), markedly reduced total hemolytic complement activity (CH50 <10 U/mL), and markedly reduced alternative pathway activity (AP50 <10 U/mL), with a normal C4 (0.18 g/L) (**Table 2**). This pattern of isolated undetectable C3 with absent functional complement activity across both pathways, and normal C4, is pathognomonic for primary complete C3 deficiency.

**Table 2:** Hematological, immunological, and complement parameters at diagnosis.

Parameter	Patient 1 (4-yr girl)	Normal range	Patient 2 (8-yr boy)	Normal range
Hematology				
Hemoglobin (g/dL)	10.8*	11.0–13.0	12.2	11.5–14.5
WBC (/mm³)	7,630	4,000–10,000	6,800	4,000–10,000
Neutrophils (/mm³)	2,620	1,500–8,000	2,924	1,500–8,000
Lymphocytes (/mm³)	4,100	1,500–4,000	3,264	1,500–4,000
Monocytes (/mm³)	430	200–1,000	476	200–1,000
Eosinophils (/mm³)	470	0–500	136	0–500
Basophils (/mm³)	10	0–100	0	0–100
Platelets (×10³/µL)	313	150–400	290	150–400
Lymphocyte immunophenotyping				
CD3+ T cells (/mm³)	2,922	1,400–3,700	2,055	1,400–3,700
CD4+ T cells (/mm³)	1,395	700–2,200	1,305	700–2,200
CD8+ T cells (/mm³)	1,217	490–1,300	646	490–1,300
CD19+ B cells (/mm³)	955	390–1,400	277	390–1,400
NK cells CD16/CD56 (/mm³)	164	130–720	400	130–720
Serum immunoglobulins				
IgA (g/L)	1.09	0.27–2.04	1.82	0.27–2.04
IgG (g/L)	9.19	5.04–15.6	5.98	5.04–15.6
IgM (g/L)	1.91	0.24–1.79	1.55	0.24–1.79
IgE (IU/mL)	94.52	<150	44	<150
Neutrophil function				
DHR oxidative burst (%)	94.23	>95	97.9	>95
Complement				
CH50 (U/mL)	<10 ↓↓	>31.6	<10 ↓↓	>31.6
AP50 (U/mL)	<10 ↓↓	>30	<10 ↓↓	>30
C3 (g/L)	<0.01 ↓↓	0.81–1.57	<0.01 ↓↓	0.81–1.57
C4 (g/L)	0.18	0.17–0.39	0.29	0.17–0.39

WBC (GB), white blood cells; CD, cluster of differentiation; Ig, immunoglobulin; DHR, dihydrorhodamine test; CH50, total hemolytic complement activity; AP50, alternative pathway hemolytic activity; C3, C4, complement components 3 and 4.

Additional familial complement investigations were recently performed in the parents of Patient 1. The mother had C3 0.7 g/L and CH50 25.4 U/mL, while the father had C3 0.65 g/L and CH50 26.1 U/mL.

The patient received pneumococcal vaccination with Prevenar 13^®^ and continuous antimicrobial prophylaxis with daily cotrimoxazole syrup (2 teaspoons/day), with a favorable clinical outcome during follow-up.

#### Patient 2

An 8-year-old boy was referred for investigation of recurrent infections and autoimmune manifestations. He originates from the same province of Taounat as Patient 1. He was born to parents with third-degree consanguinity. The pregnancy and delivery were unremarkable, with normal neonatal adaptation. He was exclusively breastfed until 7 months of age and followed the national vaccination schedule. He is the second of four siblings. His older sibling died at 9 months of age following febrile respiratory distress requiring intensive care, with no final diagnosis established. The other two siblings are clinically healthy. Family history on both parental sides includes multiple early and unexplained deaths across generations within a highly consanguineous extended family (**Figure 1A**).

Symptoms began at 1 month of age with recurrent respiratory distress. At 45 days of life, the patient underwent partial uvula resection according to traditional regional practices, a procedure that was complicated by local infection resulting in a pharyngeal abscess requiring hospitalization and parenteral antibiotic therapy. Subsequently, he experienced febrile episodes that recurred approximately every two months. From 5 months until 2 years of age, he had recurrent purulent otorrhea. The clinical course included recurrent tonsillitis, purpura episodes, a documented urinary tract infection, and intermittent headaches and ankle oedema. At 6 years of age, he was diagnosed with IgA vasculitis (Henoch-Schonlein purpura), characterized by palpable purpura on the lower extremities, ankle edema, and arthralgia, that was treated with systemic corticosteroids with good clinical response (**Table 1**).

At the time of referral, clinical examination was unremarkable, with normal growth parameters and no active signs of infection or inflammation. Immunological investigations showed normal lymphocyte subsets and immunoglobulin levels, with a normal DHR test (97.9%). Complement analysis revealed undetectable C3 (<0.01 g/L), markedly decreased CH50 (<10 U/mL), and normal C4 (0.29 g/L), confirming an isolated complete C3 deficiency (**Table 2**).

Familial complement screening was performed in both parents and siblings of Patient 2. The mother had C3 0.69 g/L and CH50 23.9 U/mL; the father had C3 0.51 g/L and CH50 28.8 U/mL.

Prophylactic treatment including Prevenar 13^®^ vaccination and daily cotrimoxazole syrup (2 teaspoons/day) was initiated, and the patient remains clinically stable under regular follow-up.

### Genetic results

Next-generation sequencing (NGS) identified a homozygous variant in the *C3* gene in both patients: c.4030-1_4030delinsCT (Chr19: g.6684661_6684662delinsAG, GRCh38/hg38). This two-nucleotide deletion-insertion replaces the canonical AG dinucleotide at the 3′ acceptor splice site of intron 31, immediately preceding exon 32, within the region encoding the C-terminal C345C domain of the α-chain (**Figure 1B**). This domain constitutes the sole binding interface on C3b for factor B, and its disruption simultaneously abolishes alternative pathway convertase assembly and C3b-mediated opsonization (6, 24). The variant is absent from all major population genetic databases, including gnomAD v4.1 (0 alleles across approximately 1,461,894 chromosomes), gnomAD v2.1.1, ExAC, and ClinVar, confirming its extreme rarity and supporting its novelty. Additionally, the C3 gene demonstrates strong constraint against loss-of-function variants in gnomAD population data, with a probability of being loss-of-function intolerant (pLI) score of 0.90, an ExAC pLI of 0.99, and a loss-of-function observed/expected upper bound fraction (LOEUF) of 0.13 (based on transcript ENST00000245907).

In silico splicing prediction tools were applied in concordance and produced unanimously damaging results. SpliceAI returned a delta score of 1.00 for loss of the canonical acceptor site, the ceiling of the scoring range indicating near-certain abolition of normal splicing, and a score of 0.72 for activation of a cryptic acceptor located 24 nucleotides upstream. Incorporation of these 24 non-triplet intronic nucleotides into the mature transcript would produce a frameshift, a premature termination codon, and consequent degradation by nonsense-mediated mRNA decay, yielding a functional null allele. Analysis through the Alamut Visual Plus pipeline, integrating SPiP and SPiCE, independently predicted an alteration probability of 98.41% with a high-impact classification. In addition, MaxEntScan, NNSPLICE, Splice Site Finder, and GeneSplicer each predicted 100% loss of the natural acceptor site. The convergence of six independent algorithms on the same outcome, each drawing on a distinct computational framework, provides robust in silico evidence for complete splice site disruption, consistent with the undetectable serum C3 documented in both patients.

Sanger sequencing was performed to confirm the NGS findings and to conduct segregation analysis. Both patients were confirmed to be homozygous for the c.4030-1_4030delinsCT variant. Parental segregation analysis, performed in the family of Patient 2, demonstrated that both the mother and father carry the variant in the heterozygous state, consistent with autosomal recessive inheritance and corroborated by their biochemical phenotype: approximately 40 to 50% of normal serum C3 with reduced but detectable CH50, consistent with heterozygous carrier status and reduced C3 protein expression Interestingly, some previously reported heterozygous parents exhibited C3 levels closer to the lower limit of normal but nevertheless developed recurrent infections (8, 25). In contrast, both parents in our study remained clinically asymptomatic despite decreased C3 and CH50 levels below the normal range, suggesting variable biological expression and incomplete clinical penetrance among heterozygous carriers. Among the patient’s two healthy siblings, one presented with borderline complement values suggesting possible heterozygous carrier status, whereas the other exhibited entirely normal parameters, likely representing homozygous wild-type genotype.

Loss-of-function (LoF) is the established and predominant disease mechanism for autosomal recessive complete C3 deficiency. In accordance with **Table 1** of the ClinGen SVI PVS1 recommendations (23), all required conditions are fulfilled: the gene-disease association for C3-associated complement deficiency holds Definitive clinical validity; at least three pathogenic LoF variants have been independently reported in ClinVar/HGMD without reliance on PVS1; LoF variants represent more than 10% of all reported pathogenic variants; and these variants are distributed across multiple exons. The near-absent serum C3 level in both patients (0.01 g/L) provides independent functional confirmation of the null effect, consistent with complete LoF, and contributes separately as functional evidence (PS3, Strong).

Applying the ACMG/AMP framework, the variant satisfies several criteria supporting a Likely Pathogenic classification. The variant NM_000064.3:c.4030-1_4030delinsCT affects the canonical splice acceptor site and is predicted to severely disrupt normal splicing. In accordance with current recommendations for splice-site variant interpretation, PVS1 was applied at a strong level (PVS1_Strong) rather than very strong, as no RNA studies were available to experimentally confirm the precise splicing consequence. The variant also fulfills PM2_Supporting, as it is absent from population databases; PP1_Supporting, based on segregation with disease within the family; PP3_Supporting, due to concordant in silico predictions indicating a deleterious effect; and PP4_Supporting, given the highly specific phenotype consistent with complete C3 deficiency. Collectively, these findings support classification of the variant as Likely Pathogenic.

### Treatment and follow-up

Following diagnosis, both patients received pneumococcal vaccination with Prevenar 13^®^ and antimicrobial prophylaxis with daily cotrimoxazole syrup (2 teaspoons/day). Patient 1 was diagnosed earlier and therefore benefited from a longer follow-up period of 6 months, whereas Patient 2 was followed for 3 months after treatment initiation. During follow-up, both patients showed favorable clinical evolution with normal growth and development and no recurrence of severe infectious episodes under prophylactic treatment. Renal monitoring was also regularly performed and remained normal in both patients.

## Discussion

We report two unrelated Moroccan pediatric patients with complete primary complement component 3 (C3) deficiency, both harboring the same homozygous splice-site variant in the *C3* gene. To our knowledge, cases from Morocco have not been documented in the literature to date. Complete C3 deficiency is an exceptionally rare inborn error of immunity, with fewer than 50 cases reported worldwide since its first description in 1972 (7, 8). The published kindreds originate from diverse ethnic and geographic backgrounds, including Dutch, South African Afrikaner, Japanese, Palestinian-Lebanese, Brazilian, Portuguese, and North American descent (10, 11) (**Table 3**). The present report adds two new cases to this limited global registry, expands the recognized clinical spectrum of the disorder, and describes a novel pathogenic variant with evidence for a founder effect in a consanguineous North African population.

**Table 3:** Comparative table of principal published cases of complete C3 deficiency, including the two patients reported in the current study.

Case reference (author, year)	Patient demographics (age, sex, ethnicity/origin)	Genetic variant (mutation details)	Clinical presentation (main symptoms)	Laboratory findings (C3, C4, immunoglobulins)	Infections/ complications	Treatment approach	Outcomes/ follow-up	Consanguinity
Current study Patient 1	4 years, Female, Moroccan (Taounat region)	NM_000064.3:c.4030-1_4030delinsCT (Homozygous) Novel splice-site variant affecting exon 32 acceptor site Encodes C345c domain	Onset: 10 months • Recurrent febrile episodes • Chronic wheezing (treated as asthma) • Recurrent purulent otorrhea • Facial cellulitis secondary to otitis media	C3: <0.01 g/L (NR: 0.81-1.57) C4: 0.18 g/L (NR: 0.17-0.39) CH50: <10 U/mL (NR: >31.6) AP50: <10 U/mL (NR: >30) IgA, IgG, IgM, IgE: Normal Lymphocyte subsets: Normal	• Recurrent respiratory infections • Recurrent otitis media with purulent otorrhea • Facial cellulitis • No meningitis or sepsis	• Vaccination: PCV13, PPSV23, Hib, MenACWY, MenB • Antibiotic prophylaxis (penicillin/amoxicillin) • Aggressive treatment of acute infections	Clinically stable at last follow-up Marked reduction in infection frequency and severity since prophylaxis initiation Attending school regularly Age-appropriate development	Yes (First-degree consanguineous parents)
Current study Patient 2	8 years, Male, Moroccan (Taounat region)	NM_000064.3:c.4030-1_4030delinsCT (Homozygous) Same novel splice-site variant as Patient 1 Suggests founder effect in Taounat region	Onset: 1 month (earlier than Patient 1) • Recurrent respiratory distress • Pharyngeal abscess (post-uvula resection at 45 days) • Recurrent purulent otorrhea (5 months-2 years) • IgA vasculitis (Henoch-Schönlein purpura) at 6 years • Recurrent tonsillitis • Urinary tract infection	C3: <0.01 g/L C4: 0.29 g/L (Normal) CH50: <10 U/mL IgA, IgG, IgM, IgE: Normal DHR test: 97.9% (Normal) Parental screening: • Mother: C3 0.69 g/L, CH50 23.9 • Father: C3 0.51 g/L, CH50 28.8	• Recurrent respiratory distress from infancy • Pharyngeal abscess • Recurrent otitis media • Recurrent tonsillitis • Urinary tract infection • Autoimmune: IgA vasculitis	• Vaccination: PCV13, PPSV23, Hib, MenACWY, MenB • Antibiotic prophylaxis • Corticosteroids for IgA vasculitis • FFP discussed but not routinely used	Stable at follow-up IgA vasculitis in remission (no recurrence of purpura or renal involvement) Decreased infection frequency Attending school regularly Family history: Elder sibling died at 9 months from febrile respiratory distress	Yes (Third-degree consanguineous parents) Multiple early deaths on both parental sides
Alper et al., 1972 [First reported case] (7)	Not specified in detail, Patient with repeated infections	Not characterized (genetic sequencing not available in 1972) Homozygous C3 deficiency confirmed biochemically	Repeated infections (specific details limited in original report)	C3: Undetectable Biochemical confirmation of homozygous C3 deficiency (Detailed immunoglobulin data not available)	Recurrent infections (Landmark first description of complete C3 deficiency)	Not detailed in available literature summary	Not available in literature summary	Not specified
Pussell et al., 1980 (19)	Palestinian-Lebanese family living in Kuwait; parents 44-year-old father, 42-year-old mother; six children aged 20, 15, 13, 12, 7½, and 5 years	Homozygous C3 deficiency in three children (specific mutation not characterized) both parents and two children heterozygous for the C3 null gene	Proteinuria and microscopic haematuria, some with hypertension, abdominal pain, rash; one with nephrotic syndrome and mesangiocapillary glomerulonephritis; recurrent respiratory/ENT symptoms	C3 very low or absent in three homozygous children; reduced in three heterozygotes; C4 reported as within normal range; immunoglobulin levels normal or high in all except slight IgG reduction in the daughter with membranoproliferative GN	All homozygous and heterozygous C3-deficient children had some susceptibility to infection; documented osteomyelitis (father), frequent respiratory infections, earache, sore throat; pneumococcal peritonitis in one homozygous child, previous peritonitis in another; nephritis in several children	Corticosteroids for membranoproliferative glomerulonephritis; prednisone and cyclophosphamide for nephritis/hypertension in index child; penicillin for pneumococcal peritonitis; antihypertensive treatment	Index girl remained well after treatment for hypertension until episode of pneumococcal peritonitis, which responded to penicillin;	Probably yes – authors note “It seems likely that the parents are cousins” based on HLA/C3 data
Sano et al., 1981 (26)	Two sisters Patient 1: 19-year-old Patient 2: 14–15-year-old Ethnicity: Japanese	Hereditary C3 deficiency (specific mutation not characterized in 1981)	Systemic lupus erythematosus (SLE)-like symptoms: • Skin manifestations • Arthralgias • Fever • Autoimmune features	C3 not detected immunochemically in both; CH50 very low (6.3 and 5.8, vs 40 normal); C4 protein reduced (11.7 mg/dL; normal 13–23); IgG elevated (2320 and 2560 mg/dL), IgA and IgM normal-high Presence of autoantibodies and SLE-like serological profile	Not primary feature; autoimmune manifestations predominant	Likely immunosuppression for SLE-like disease Both received prednisone	Lupus-like manifestations improved partially with steroids	Not specified
Borzy et al., 1988 (14)	10-year-old Laotian boy	Inherited C3 deficiency (specific mutation not characterized)	• Recurrent bacterial infections (meningitis, pneumonia) • Glomerulonephritis	C3: Deficient Renal biopsy findings consistent with glomerulonephritis	Reccurent pyogenic bacterian infections (meningitis-pneumonia) glomerulonephritis	Antibiotics for infections, prophylactic sulfamethoxazole-trimethoprim, single infusions of fresh normal plasma (FFP) during pneumonia episodes	Normal growth and development	Not specified
Botto et al., 1992 (17)	Patient with homozygous C3 deficiency Age and sex not specified in summary	C3 gene: Partial gene deletion Deleted region: Exons 21-23 (Large deletion mutation)	Recurrent severe infections	C3: Undetectable due to gene deletion Absent C3 protein production	Severe recurrent infections characteristic of C3 deficiency	Not detailed	Not available	Yes (first cousins)
Singer et al., 1994 (12)	Patient with inherited C3 deficiency Age/sex not specified	C3 gene: Missense mutation in β-chain Asp549Asn (Aspartic acid to Asparagine at position 549) Protein produced but secretion impaired	Recurrent pyogenic infections due to functional C3 deficiency despite protein synthesis	C3: Very low in serum (intracellular retention) Protein synthesized but not secreted into circulation	Recurrent infections due to absence of functional circulating C3	Not detailed	Not available	Not specified
Katz et al., 1995 (15)	Patient with compound heterozygous C3 deficiency Age/sex not specified	C3 gene: Compound heterozygous Two different pathogenic variants (one on each allele) Specific mutations not detailed in summary	Clinical features of C3 deficiency	C3: Markedly reduced Compound heterozygosity confirmed by genetic analysis	Recurrent infections	Not detailed	Not available	Unlikely (compound heterozygous suggests different parental variants)
Matsuyama et al., 2001 (27)	36 y.o woman Patient 1: 36-year-old woman; Patient 2: 34-year-old woman; Japanese sisters inferred from authors/institutions	Homozygous C3 stop codon, exon 26, C3303G (Tyr1081Stop); mother and brother heterozygous with half-normal C3	Both developed SLE-like symptoms in adolescence: high fever, butterfly rash, Raynaud’s phenomenon, intermittent arthralgia, photosensitivity	C3: not detected immunochemically in both; CH50 markedly reduced (6.3 and 5.8 U/mL vs 40 normal); C4 protein 11.7 (reported as normal pool value); other complement proteins mostly normal or high; C3 activity very low (0.6–0.7% of normal)	Main complications: autoimmune, SLE-like disease (no recurrent severe infections noted in this report; previous paper described them as “SLE-like symptoms”)	Corticosteroid hormone therapy with relatively good symptom control	Symptoms controlled on steroids; mother and brother healthy carriers with half-normal C3; follow-up not otherwise detailed	Not explicitly stated; parents’ relationship not described, so consanguinity unknown
Reis et al., 2002 (28)	Proband CA, 19-year-old male, Brazilian; consanguineous Brazilian family	Homozygous nonsense mutation in C3: G→A at nucleotide 1716 in exon 13, changing Lys552 codon to stop (TGG→TAG); also a silent 972G→A (Ala304) and polymorphic L314P (T1001C)	History of recurrent and severe infections: bronchopneumonia, meningococcal meningitis, otitis media, osteomyelitis, pyodermitis, urinary tract infection, arthritis, fever of unknown origin	Serum C3 undetectable by ELISA and radioimmunoassay; no complement-dependent opsonins or hemolytic activity in serum; markedly reduced C3 mRNA (~20-fold less than control) in LPS-stimulated fibroblasts	Recurrent severe pyogenic infections (Neisseria meningitidis specifically mentioned as meningococcal meningitis); high morbidity typical of complete C3 deficiency	Article focuses on molecular characterization; specific long-term prophylactic or acute treatments are not detailed in the excerpt	Clinical course: alive at 19 years with history of multiple severe infections; two clinically healthy brothers; one brother died at 5 months after prolonged diarrhea	Yes – parents are first cousins; additional consanguinity through maternal great-grandparents
Tsukamoto et al., 2005 (18)	22-year-old Japanese male	Homozygous deletion of exon 39 (84 bp) due to a novel AG → GG 3’-splice acceptor site mutation in intron 38, leading to defective C3 secretion	Systemic lupus erythematosus (SLE), photosensitivity, recurrent fever, facial erythema, tonsillitis, recurrent pneumonia, butterfly rash, leukopenia, positive antinuclear antibody (ANA), and steroid psychosis	C3: Undetectable or very low Autoantibodies positive SLE serological profile	Recurrent tonsillitis and pneumonia, steroid psychosis SLE as prominent feature	Immunosuppression for SLE Infection prophylaxis	Not available	Yes (consanguineous marriage)
Kida et al., 2008 (29)	2-year-old Japanese boy	Compound heterozygous C3: 3176insT in exon 24 (frameshift, K1105X) + C3303G (Y1081X) in exon 26	Recurrent bacterial infections: meningitis, bronchitis, pneumonia	Undetectable serum C3 (<2 mg/dL); low CH50 (<12 U/mL); C4 normal; parents and sister with reduced/normal C3 and normal C4	Severe pyogenic infections (meningitis, pneumonia, bronchitis)	Not detailed in excerpt (likely infection-directed care	Not available	No consanguinity
Goldberg et al., 2011 (30)	4-year-old boy, Arab descent, Palestine	Homozygous single adenine deletion in exon 31 of C3 cDNA (3997delA) causing premature stop 13 codons later	Three episodes of invasive pneumococcal disease with high fever and ill appearance; two episodes with pneumonia on chest X-ray	C3 antigen <10 mg/dL (normal 90–180); C4–280 mg/L (normal 93–380); factor B, H, I and CD46 normal; IgG 990 mg/dL, IgM 155 mg/dL, IgA 154 mg/dL (all within normal ranges)	Recurrent invasive pneumococcal septicemia; pneumococcal serotypes 10B, 14, 29 isolated from blood over 20 months	Sequential vaccination: PCV-7 (Prevnar) then PPV (Pneumovax); evaluation of antibody responses; no C3 replacement reported		Yes – parents first cousins (consanguineous marriage)
Okura et al., 2011 (31)	4-year-old Japanese boy	Compound heterozygous C3 mutations: nonsense 1432C>T (Arg478Ter) in exon 12 (maternal) and splice-site IVS9 −2 a>t (paternal)	Recurrent infections: two episodes of pneumococcal bacteremia, pneumonia, acute otitis media, gastroenterocolitis	8C3 extremely low 0.3 mg/dL (N 75–150); CH50 4.7 U/mL (N 35–45); C4 normal; parental and sibling C3 mildly low/normal, CH50 near normal	Severe invasive bacterial infections: recurrent Streptococcus pneumoniae bacteremia and pneumonia; later, RF-positive synovitis of knee at 6 years (mono-arthritis)	Synovitis episode treated with naproxen initially, then methotrexate plus prednisolone; later maintained with naproxen alone	Good response of arthritis; improvement clinically and biologically;	Parents non-consanguineous in genetic report
Santos-Valente et al., 2013 (32)	16-year-old male, Turkish, third of eight children	Homozygous missense mutation in C3: c.C4554G, p.Cys1518Trp in C345C domain; both parents carriers	Recurrent lower respiratory infections from infancy; pneumonia, pleural effusion at 6 months (thoracostomy), bronchiectasis detected at 8 years, multiple hospitalizations for lower respiratory tract infections; episodes of otitis media;	C3 severely decreased: 8–19 mg/dL (normal 90–180); CH50 = 0; C4 normal (20 mg/dL); other complement components normal; IgA markedly reduced (<5.8 mg/dL); IgG, IgM, IgE and IgG subclasses within normal ranges	Recurrent pneumonias due to Streptococcus pneumoniae; bronchiectasis; otitis media; two brothers died in neonatal period (unspecified cause)	Pneumococcal polysaccharide vaccination (PNEUMO 23); assessment of specific anti-pneumococcal IgG response	Marked clinical improvement after vaccination; no severe infections or hospitalizations during 7-year follow-up; good vaccine antibody response	Yes – parents first-degree cousins (consanguineous)
El Sissy et al.,2019 (33)	4 patients (3 boys,1 girls) (7–11 y.o)	Not available	Infectious disease Pneumococcal infection	Decreased C3	–	–	–	–
Coelho et al., 2022 (13)	Pediatric patient with recurrent pyogenic infections Age/sex not specified in summary	C3 gene: Novel nonsense mutation Gln1420* (Glutamine→Stop codon at position 1420) Premature termination codon Truncated protein	Recurrent pyogenic infections: • Severe bacterial infections • Infections with encapsulated organisms	C3: Undetectable or very low CH50: Markedly reduced AP50: Markedly reduced	Recurrent severe pyogenic infections (primary presentation)	Vaccination against encapsulated bacteria Antibiotic prophylaxis	Not detailed in summary	Not specified
Bernacchia et al., 2024 (8)	Two siblings Age/sex not specified in detail Ethnicity: Not specified	C3 gene: Compound heterozygous mutations Two different novel pathogenic variants Specific mutations not detailed in summary	Sibling 1: • Severe recurrent infections (pneumonia, sinusitis, osteomyelitis) • Hemolytic uremic syndrome (HUS) with acute kidney injury Sibling 2: • Severe recurrent infections • Less severe overall course	C3: Total deficiency CH50: Absent AP50: Absent Associated humoral defects: • Decreased memory B cells • Hypogammaglobulinemia • Impaired response to polysaccharide antigens	• Recurrent pneumonia • Sinusitis • Osteomyelitis • HUS in one sibling (severe complication)	• Intravenous immunoglobulin (IVIG) replacement therapy (for associated hypogammaglobulinemia) • Vaccination • Antibiotic prophylaxis	Variable between siblings despite same genetic defect Demonstrates phenotypic heterogeneity One sibling developed life-threatening HUS	Not specified (compound heterozygous suggests different parental variants)

The clinical phenotype of C3 deficiency, as established in early case reports, is dominated by severe, life-threatening recurrent pyogenic infections, particularly meningitis, sepsis, and pneumonia caused by encapsulated bacteria such as *Streptococcus pneumoniae*, *Haemophilus influenzae*, and *Neisseria meningitidis*, reflecting the indispensable role of C3b-mediated opsonization in defense against encapsulated organisms (2, 7, 10, 11, 19, 34). Neither of our patients fits this classical severe infectious phenotype: neither sustained documented bacteremia, meningitis, or deep tissue infection. Instead, both experienced chronic, recurrent infections primarily restricted to the upper respiratory tract and middle ear. Such phenotypic variability has been described in previously published kindreds, where patients carrying identical mutations followed markedly divergent courses (8, 14). The molecular basis for this clinical variability is incompletely understood but likely reflects the contribution of modifier alleles in complement regulatory genes, differences in pathogen exposure, microbiome composition, and stochastic immunological events.

The development of autoimmune and immune complex-mediated disease is a recognized feature of C3 deficiency: approximately 26% develop SLE-like autoimmune illness and around 26% develop membranoproliferative glomerulonephritis, attributable to failure of C3-dependent immune complex solubilization and clearance (9, 11, 26, 27, 35). The occurrence of IgA vasculitis (IgAV) in both patients falls within this same pathophysiological framework. While vasculitis manifestations have been previously documented in C3 deficiency (36), IgAV as a distinct clinicopathological entity has not, to our knowledge, been previously described in this setting (**Table 3**). In the absence of C3b, circulating IgA1-containing immune complexes accumulate and deposit in small vessel walls, where they drive vascular inflammation through complement-independent mechanisms including Fc receptor engagement, cellular recruitment, and direct endothelial activation (9, 37). Impaired clearance of apoptotic cellular debris, which also depends on C3-mediated opsonization, may further contribute to the breach of self-tolerance observed in affected patients (9, 37). The autoimmune predisposition in C3 deficiency underscores the dual role of complement in both host defense against pathogens and maintenance of immunological self-tolerance.

From a molecular standpoint, our identification of the novel homozygous splice-site variant c.4030-1_4030delinsCT in both patients expands the mutational spectrum of C3 deficiency. In silico predictions uniformly indicated complete loss of the natural splice site with likely frameshift and nonsense-mediated decay, consistent with the undetectable C3 protein levels observed clinically. This variant is located at the canonical acceptor site of exon 32, within the region encoding the C345C domain of the C3 α-chain. This domain is the sole binding site on C3b for factor B and is therefore essential for assembly of the alternative pathway C3 convertase (24, 38). Splice-site variants in the *C3* gene are not uncommon: among the 15 characterized mutations in a systematic review, 6 were splicing abnormalities (10). However, the only homozygous *C3* splice-site mutation causing C3 deficiency and concurrent autoimmune disease was reported in a Japanese patient with homozygous C3 deficiency and SLE-like disease, who carried a mutation at intron 38 (18). In this context, the homozygous splice-site variant identified in our patients expands the mutational spectrum of C3 deficiency and strongly supports a loss-of-function mechanism.

The location of a pathogenic variant within the C3 protein appears to influence the clinical phenotype, as illustrated by the domain-by-domain comparison in **Table 4**. In patients whose mutations are situated in the β-chain or upstream of the thioester-containing domain (TED), regions encoding the N-terminal half of the protein (MG1–MG7, CUB), the predominant manifestation is severe, recurrent bacterial infection, consistent with large truncations or complete absence of the C3 protein (10). By contrast, mutations concentrated within or downstream of the TED domain, and in particular those affecting the C345C domain, have been associated with milder infectious phenotypes, immune-complex–mediated disease, or both (10). The C345C domain is of particular structural relevance to the present report. This domain constitutes the sole binding interface on C3b for factor B, and its disruption simultaneously abolishes alternative pathway convertase assembly and C3b-mediated opsonization. Among the cases reviewed, three previously published mutations directly affect the C345C domain, each associated with a distinct clinical expression.

**Table 4:** Published C3 deficiency cases with confirmed molecular analysis.

Patient	Age/sex	C3 Variant	Domain	Clinical features	Reference
1	15y/F	800-bp deletion (homo)	MG1-2 (β-chain)	Meningitis ×3, pneumonia ×14, recurrent otitis media, paronychia, impetigo, Sweet syndrome	(7, 17)
2	10y/F	IVS10 + 1g>t (homo)	MG5 (intron 10)	Pneumonia ×12, otitis media ×5, septic arthritis, buttock abscess	
3	6y/M	G1655A: p.Lys552X (homo)	MG6	Meningitis ×3, pneumonia ×4, otitis media ×4, osteomyelitis ×2, skin infections, arthritis	(28)
4	10y/M	IVS18 + 1g>a (homo)	CUB (intron 18)	Recurrent otitis media, >20 episodes of erythematous plaques	(39)
5	8y/M	C2542T: p.Arg848X (homo)	MG7/TED	Lymphadenitis, sinusitis ×2, pneumonia, tonsillitis ×2, giardiasis	(40, 41)
6	4y/M	3997delA (homo)	TED	Pneumonia ×2, bacteremia ×3	(30)
7	16y/M	C4554G: p.Cys1518Trp (homo)	C345C	Recurrent pneumonia, otitis media, bronchiectasis, concomitant IgA deficiency	(32)
8	2y/M	3176dupT + C3243G: p.Tyr1081X (hetero)	TED/LNK	Meningitis, bacteremia ×4, pneumonia ×2, otitis media, focal segmental glomerulonephritis, SLE-like illness	(29)
9	4y/M	IVS9 -2a>t + C1432T: p.Arg478X (hetero)	MG5/MG6	Bacteremia ×2, pneumonia ×2, otitis media, synovitis	(31)
10	4y/M	IVS11 + 5g>a + IVS12 -1g>t (hetero)	MG6	Bacteremia, otitis media, sinusitis, membranous nephropathy, SLE-like illness	(10)
11-1 11-2	19y/F; 14y/F	C3243G: p.Tyr1081X (homo)	LNK	SLE-like illness — no severe infections	(26, 27)
12	23y/M	IVS38 -2a>g (homo)	C345C	Recurrent tonsillitis and pneumonia in late teens; SLE-like illness at age 20 — mild infections	(18)
13	7y/M	3736_3737delTT (homo)	C345C	Bronchitis, otitis media, membranous nephropathy — no severe infections	(42)
Current patients	4y/F 8y/M	c.4030-1_4030delinsCT (homo)	C345C (splice)	Reccurent febrile episode—Recuurent respiratoey distress—Reccurent purulent otorrhea	Present report

homo, homozygous; hetero, heterozygous; SLE, systemic lupus erythematosus. Adapted from Okura et al., J Allergy Clin Immunol 2016.

A particularly noteworthy aspect of our report is the strong evidence for a founder effect in the Taounat region of Morocco. The occurrence of two unrelated patients from the same geographically confined area, both carrying an identical novel homozygous variant that is absent from all global population databases, in the context of documented consanguinity in both families, constitutes compelling evidence for a founder mutation. Founder effects occur when a rare variant is introduced into a population by a common ancestor and becomes enriched in that population, particularly when consanguineous marriage is common (20, 21). The Middle East and North Africa (MENA) region, including Morocco, has particularly high rates of consanguineous marriage, with parental consanguinity reported in 60.5% of families with inborn errors of immunity in the MENA Registry (20). Founder effects are well-characterized in autosomal recessive IEIs in populations with elevated consanguinity rates (21, 43, 44), and the province of Taounat lies within a region of Morocco characterized by a sustained tradition of endogamous marriage. Formal haplotype analysis using flanking microsatellite markers or SNP arrays would be required to definitively confirm identity by descent.

These cases also carry important practical diagnostic implications. In both patients, the correct diagnosis was reached only after complement testing was performed, which occurred several years after symptom onset. This delay is not uncommon in complement deficiency and underscores the need to include complement screening, specifically serum CH50, AP50, C3, and C4, in the standard immunological evaluation of any child presenting with recurrent infections of unexplained etiology, particularly in Morocco and similar resource-limited settings, even when the infections appear moderate or are restricted to the upper respiratory tract, even when the infections appear moderate or are restricted to the upper respiratory tract. The pattern of undetectable C3 with normal C4 and absent CH50/AP50 is pathognomonic for C3 deficiency and should trigger immediate referral for genetic analysis (3, 5). Moreover, these cases highlight that autoimmune manifestations, including IgAV, should be recognized as part of the clinical landscape and the complement system should be evaluated not only in infectious but also in inflammatory contexts. Genetic confirmation through NGS not only establishes the molecular diagnosis but also enables accurate genetic counseling.

The management of both patients was guided by the ESID/ERN RITA Complement Guideline, recommending a multifaceted approach centered on infection prevention and prompt treatment (2, 3). Comprehensive vaccination against encapsulated organisms (pneumococcal, Haemophilus influenzae, meningococcal) is also recommended (2), though efficacy may be reduced given the role of complement in optimal antibody responses, particularly to polysaccharide antigens (8, 10). In C3-deficient cases with associated hypogammaglobulinemia, intravenous immunoglobulin (IVIG) replacement may be considered to restore humoral protection (8). Continuous antibiotic prophylaxis, typically with penicillin, significantly reduces infection frequency and is strongly recommended (2, 3). Education of families regarding the need for emergency evaluation of any febrile illness is critical, as progression to sepsis can be rapid (3). Fresh frozen plasma (FFP) infusions can provide temporary complement replacement during acute infections or perioperative periods, though the logistical challenges, risks, and short duration of effect limit its routine prophylactic use (2). Autoimmune complications require individualized treatment, typically involving immunosuppressive agents, though this must be carefully balanced against the underlying immunodeficiency (2, 3).

This report has several limitations. First, functional validation of the splice-site variant at the mRNA and protein level was not performed. RNA analysis by RT-PCR on patient-derived cells would have directly confirmed the predicted incorporation of intronic nucleotides into the mature transcript and the resulting frameshift, while western blot or C3 protein expression studies would have corroborated the absence of functional C3 protein. Although the convergent in silico predictions, undetectable serum C3, and clinical phenotype together provide strong inferential evidence for pathogenicity, direct demonstration of aberrant splicing would strengthen the classification to Pathogenic. Furthermore, extended familial genetic segregation analyses were limited by financial constraints and the inability to obtain blood samples from certain family members for additional genetic investigations. Formal segregation analysis was not performed in the parents of Patient 1 because blood samples for genetic testing could not be obtained from the family. Similarly, although biochemical complement screening was conducted in the siblings of Patient 2, genetic testing could not be extended to asymptomatic relatives, including the sibling with borderline complement values suggestive of heterozygous carrier status, because blood samples for molecular analysis were not obtained and financial limitations precluded further investigations. Additional family studies would have provided stronger segregation evidence supporting the pathogenic role and inheritance pattern of the identified variants. Second, we did not obtain kidney biopsies or detailed renal function studies in either patient to comprehensively assess for subclinical glomerular disease, which has been frequently reported in C3 deficiency (14, 19). Third, the long-term outcomes and efficacy of the implemented management strategies in our patients remain to be determined through extended follow-up. Fourth, segregation analysis was performed only in the family of Patient 2. Finally, formal haplotype analysis to confirm a founder effect was not conducted in the Taounat region. Despite these limitations, the comprehensiveness of the clinical and genetic characterization and the novelty of both the variant and the phenotypic observations sustain the clinical and scientific value of this report.

## Conclusion

In conclusion, we report two patients with complete C3 deficiency associated with a novel homozygous splice-site mutation affecting a critical functional domain of the protein. To the best of our knowledge, this variant has not been previously reported. Notably, our findings expand both the clinical and genetic spectrum of C3 deficiency and highlight that the disease may present with non-severe but chronic and recurrent infections. These observations emphasize the importance of considering complement deficiencies in atypical clinical contexts and support the integration of genomic approaches into the diagnostic workflow of inborn errors of immunity.

## Data Availability

The raw data supporting the conclusions of this article will be made available by the authors upon reasonable request, subject to applicable ethical and privacy considerations.
